# Adipose Tissue-Derived Mesenchymal Stem Cells in Long-Term Dialysis Patients Display Downregulation of PCAF Expression and Poor Angiogenesis Activation

**DOI:** 10.1371/journal.pone.0102311

**Published:** 2014-07-15

**Authors:** Shuichiro Yamanaka, Shinya Yokote, Akifumi Yamada, Yuichi Katsuoka, Luna Izuhara, Yohta Shimada, Nobuo Omura, Hirotaka James Okano, Takao Ohki, Takashi Yokoo

**Affiliations:** 1 Division of Regenerative Medicine, Department of Internal Medicine, The Jikei University School of Medicine, Tokyo, Japan; 2 Division of Nephrology and Hypertension, Department of Internal Medicine, The Jikei University School of Medicine, Tokyo, Japan; 3 Department of Pediatrics, The Jikei University School of Medicine, Tokyo, Japan; 4 Department of Gene Therapy, Institute of DNA Medicine, The Jikei University School of Medicine, Tokyo, Japan; 5 Department of Surgery, The Jikei University School of Medicine, Tokyo, Japan; IRCSS - Istituto di Ricerche Farmacologiche Mario Negri, Italy

## Abstract

We previously demonstrated that mesenchymal stem cells (MSCs) differentiate into functional kidney cells capable of urine and erythropoietin production, indicating that they may be used for kidney regeneration. However, the viability of MSCs from dialysis patients may be affected under uremic conditions. In this study, we isolated MSCs from the adipose tissues of end-stage kidney disease (ESKD) patients undergoing long-term dialysis (KD-MSCs; mean: 72.3 months) and from healthy controls (HC-MSCs) to compare their viability. KD-MSCs and HC-MSCs were assessed for their proliferation potential, senescence, and differentiation capacities into adipocytes, osteoblasts, and chondrocytes. Gene expression of stem cell-specific transcription factors was analyzed by PCR array and confirmed by western blot analysis at the protein level. No significant differences of proliferation potential, senescence, or differentiation capacity were observed between KD-MSCs and HC-MSCs. However, gene and protein expression of p300/CBP-associated factor (PCAF) was significantly suppressed in KD-MSCs. Because PCAF is a histone acetyltransferase that mediates regulation of hypoxia-inducible factor-1α (HIF-1α), we examined the hypoxic response in MSCs. HC-MSCs but not KD-MSCs showed upregulation of PCAF protein expression under hypoxia. Similarly, HIF-1α and vascular endothelial growth factor (VEGF) expression did not increase under hypoxia in KD-MSCs but did so in HC-MSCs. Additionally, a directed *in vivo* angiogenesis assay revealed a decrease in angiogenesis activation of KD-MSCs. In conclusion, long-term uremia leads to persistent and systematic downregulation of PCAF gene and protein expression and poor angiogenesis activation of MSCs from patients with ESKD. Furthermore, PCAF, HIF-1α, and VEGF expression were not upregulated by hypoxic stimulation of KD-MSCs. These results suggest that the hypoxic response may be blunted in MSCs from ESKD patients.

## Introduction

The number of end-stage kidney disease (ESKD) patients is increasing worldwide [1]. Dialysis therapy for ESKD results in heavy physical and mental burdens, and associated annual medical expenses are very high [2]. Development of a treatment method that does not involve dialysis is therefore desirable to reduce expenses and increase the quality of life of patients. Kidney transplantation significantly prolongs the life expectancy of chronic kidney disease (CKD) patients [3], [4] and is less expensive than dialysis, but there is a shortage of organs available for transplantation, and lifetime immunosuppressant therapy is required for patients [5].

This critical shortage of organs has driven new technologies such as tissue engineering and regenerative medicine to achieve functional kidney replacement [5], [6]. Our previous studies showed that a xenobiotic developmental process for growing xenoembryos allows exogenous human mesenchymal stem cells (MSCs) to undergo epithelial conversion and form a nephron that produces urine and erythropoietin [7]–[9]. These findings suggested that MSCs might be a cell source for future renal regeneration. Furthermore, MSCs are easy to obtain in large numbers and are not costly to establish [10], [11].

Previously, we used bone marrow-derived MSCs from healthy volunteers, although it is unclear whether these differ from MSCs from dialysis patients. This is because patients with terminal renal failure have been exposed to uremic toxins over long periods, which may affect the viability and regenerative capacity of MSCs, suggesting that they may be unsuitable for kidney regeneration. Similarly, some reports have suggested that the regenerative capacity of adult stem cells in patients with chronic renal failure is inferior to those in patients with normal renal function [12], [13]. However, a recent report found that adipose tissue-derived MSCs (ASCs) of patients with renal disease have similar characteristics and functionality to those from healthy individuals in terms of their immunosuppressive capacities, and expression of pro-inflammatory and anti-inflammatory factors [14]. Despite these findings, the previous report did not analyze the expression of stemness genes in ASCs.

Previously, we evaluated the differentiation capabilities and gene expression profiles of bone marrow-derived MSCs and ASCs from normal rats and those with adenine-induced renal failure [15]. Although the uremic toxin has only a small effect on the gene expression and differentiation of MSCs, we used a rat model of CKD and the exposure time to the toxin was shorter than in human ESKD because of the short lifespan of the rat. Actual ESKD patients have a much longer duration of renal insufficiency.

In this study, to clarify the effect of long-term CKD on ASCs, we explored differences in the expression profiles of stemness and other important genes in ESKD patients (KD-MSCs) and healthy controls (HC-MSCs) using RT-PCR array analysis. We hypothesized that downregulation of p300/CBP-associated factor (PCAF) in the long-term uremic state might render KD-MSCs as an inappropriate cell source for kidney regenerative therapy.

## Materials and Methods

### Ethics Statement

This study was conducted according to the principles of the Declaration of Helsinki and approved by the Ethics Committee of The Jikei University School of Medicine. All donors provided written informed consent for collection of samples and subsequent analyses.

### Isolation and Culture of Human MSCs

Patient characteristics are shown in Table 1 (glomerular filtration rate was calculated using the modification of diet in the renal disease study equation). Subcutaneous or mesenteric adipose tissue was surgically removed from ESKD patients (*n* = 9, mean age: 52.6 years; mean glomerular filtration rate: 4 ml/min/1.73 m^2^) and healthy controls (*n* = 6, mean age: 56.2 years; mean glomerular filtration rate: 73.5 ml/min/1.73 m^2^). All ESKD patients were undergoing dialysis (CKD stage 5D). Experiments were independently performed for each donor.

**Table 1:** Characteristics of patients at time of adipose tissue sampling.

	Healthy controls (n = 6)	End-stage kidney disease patients (n = 9)
Sex (F/M)	2/4	2/7
Age, years (mean, range)	56.2 (50–63)	52.6 (37–64)
BMI (mean, range)	23.5 (18.4–27.3)	22.6 (17.0–30.1)
Creatinine, mg/dl (mean, range)	0.79 (0.53–1.01)	11.9 (6.8–16.5)
eGFR, ml/min per 1.73m2 (mean, range)	73.5 (62–89)	4.0 (3–6)
Duration of RRT (months, range)		72.3(±26.3)
Diabetes mellitus	1/6	4/9
Hypertension	5/6	7/9
Medication		
Anti-hypertensive drugs	5/6	7/9
Erythropoiesis-stimulating agents	NA	7/9
Anti-platelet drugs	0/6	2/9
Cholesterol-lowering drugs	1/6	2/9
Prednisone	0/6	0/9
Insulin	0/6	3/9
KTx indication		
RPGN		1
IgA nephropathy		1
Adult-onset polycystic kidney disease		1
Diabetic nephropathy		4
von Gierke's disease		1
unknown		1

Tissues were placed in Hanks' balanced salt solution (Gibco Life Technologies, Grand Island, NY) with 100 IU/ml penicillin and 100 mg/ml streptomycin (Gibco Life Technologies). MSCs were isolated from the adipose tissues of all 15 participants by a previously described culture method [16], [17] and used for KD-MSCs (ESKD patients; *n* = 9) and HC-MSCs (healthy controls; *n* = 6). After mincing and washing with phosphate-buffered saline (PBS) (Gibco Life Technologies), the adipose tissues were enzymatically dissociated with 1 ml of 0.1% collagenase (type I) (Wako, Osaka, Japan) in PBS for 1 h at 37°C. The dissociated tissue was combined with 8 ml α-minimum essential medium (Gibco Life Technologies) supplemented with 20% fetal bovine serum (FBS) (Invitrogen, Carlsbad, CA) and then centrifuged at 300 *g* for 5 min at room temperature. After washing with PBS, the isolated adipose cells including MSCs were cultured in α-minimum essential medium supplemented with 20% FBS to prevent the inclusion of serum from renal disease patients. MSCs of passage numbers 3–5 were analyzed after 14–28 days following isolation from adipose tissue.

### Flow Cytometric Analysis of MSCs

The International Society for Cell Therapy previously suggested the following minimal criteria to define human MSCs [18]: expression of CD105, CD73, and CD90, and no expression of CD45, CD34, CD14, CD11b, CD79α, CD19, or HLA-DR surface molecules. Cells were harvested by treatment with 0.05% trypsin-EDTA (Sigma-Aldrich, St Louis, MO) for 3 min at 37°C, recovered by centrifugation at 400× *g* for 5 min, washed twice in ice-cold PBS containing 2% FBS, and re-suspended at 1×10^5^ cells/antibody test. The expression of specific MSC markers was assessed using the following antibodies: CD14-FITC, CD31-PE, CD105-FITC, CD34-PE, CD45-PE, CD73-PE, and CD90-APC (all purchased from Abcam, Cambridge, UK). Negative control staining was performed using FITC/PE/APC-conjugated mouse IgG_1_ isotype antibodies (Abcam). After incubation for 30 min at room temperature in the dark, the cells were washed with PBS, resuspended in 100 µL PBS, and analyzed by a MACSQuant flow cytometer (Miltenyi Biotec, Gladbach, Germany).

### Senescence-associated β-galactosidase (SA-β-gal) Staining and Cell Proliferation Assay

The senescence assay was performed using a senescence β-galactosidase staining kit (Cell Signaling Technologies, Danvers, MA) according to the manufacturer's instructions. Cells from passages 5, 8, and 10 were observed under a light microscope (Nikon, Tokyo, Japan) for blue coloration, and a minimum of 100 cells were counted in 10 random fields to determine the percentage of β-galactosidase-positive cells [19], [20].

Senescence-associated beta-heterochromatic foci (SAHF) analysis was performed by culturing cells and fixing them with 4% paraformaldehyde (Wako). After washing with PBS, cells were permeabilized with 0.2% Triton X-100 (Nacalai Tesque, Kyoto, Japan)/PBS for 10 min. DNA was visualized after DAPI (1 µg/ml) staining (Life Technologies) for 1 min, and washing twice with PBS as previously described [20]. DAPI-stained MSCs were observed under a light microscope (Nikon).

Proliferation rates of MSCs were determined by counting cell numbers and calculating population doubling (PD). Cells were cultured in 60-mm tissue culture dishes at 2×10^4^ cells/dish. At confluency, they were trypsinized and counted by a cell counter (Luna automated cell counter; Logos Biosystems, Gyunggi, Korea). At passages 5–10, PD was determined by the formula: PD = [log10(NH)−log10(NI)]/log10(2) where NI is the initial cell number and NH is the cell number at harvest [21]. The cumulative PD level was the sum of PDs in culture. The mean and SD were calculated from three independent experiments. Statistical analysis was carried out using a *t*-test. *P*-values of less than 0.05 were considered significant. All senescence assay measurements were performed in duplicate.

### Induction of Adipogenesis, Osteogenesis, and Chondrogenesis

Passage 3–5 MSCs were trypsinized and re-seeded in induction medium with various hMSC differentiation Kits (Poietics human mesenchymal stem cells; Lonza, Walkersville, MD) for adipogenic, osteogenic, or chondrogenic induction. MSCs were maintained in culture according to the manufacturer's protocols.

### Detection of Adipogenesis and Osteogenesis

Adipogenic and osteogenic differentiation of MSCs were evaluated by measuring glycerol-3-phosphate dehydrogenase (GPDH) and alkaline phosphatase (ALP) activity, respectively. The GPDH assay kit (MK426) was obtained from Takara Bio (Shiga, Japan), and the ALP assay kit was from Wako. Assay plates were analyzed using a microplate reader (SH-1000; Hitachi High-Technologies, Tokyo, Japan). GDPH and ALP activities were normalized to the protein concentration determined by a DC protein assay kit (Bio-Rad, Hercules, CA).

### Histopathological Examination

Adipocytes differentiated from MSCs were stained with Sudan III. Osteoblasts differentiated from MSCs were stained by the von Kossa method. Chondrocytes differentiated from MSCs were stained with Safranin O, Fast green, and Toluidine blue using a Cartilage Staining Kit (Takara Bio). The cells were photographed under a microscope.

### RT-PCR Array Analysis

Gene expression profiles of stem cell-specific transcription factors of MSCs at passages 3–5 were analyzed by RT-PCR Array (PAHS-082ZA; Qiagen, Hilden, Germany) in accordance with the manufacturer's recommendations of 84 key genes and five housekeeping genes (Table 2). Briefly, total RNA was extracted using an RNeasy Mini Kit (Qiagen), and 1 µg total RNA was used to generate cDNA (First Strand Kit, Qiagen). Real-time PCR was performed using an ABI 7300 Real-time PCR System (Applied Biosystems, Foster City, CA) with RT2 SYBR Green qPCR Master mix (Qiagen) and a Human Mesenchymal Stem Cell PCR Array (Qiagen) according to the manufacturer's instructions. Briefly, the cDNA was diluted and mixed with an equal amount of SYBR Green Master mix, which was previously aliquoted (25 µl) into each well of a 96-well PCR array plate containing predispensed gene-specific primer sets. PCR was then performed according to the manufacturer's instructions. The thermal cycling conditions were: 95°C for 10 min, followed by 45 cycles of 95°C for 15 s then 56°C for 1 min. Data (fold changes in C_t_ values of all genes) were analyzed using Qiagen software. *P*-values were calculated based on the Student's *t*-test of replicate 2∧(−ΔCt) values for each gene in control and treatment groups.

**Table 2:** Differences in mRNA expression between KD-MSCs and HC-MSCs by RT-PCR analysis.

GeneBank No.	Gene Name	Gene Symbol	Fold Change	T-Test	
			KDMSC/HCMSC	p Value	* p<0.05
Stemness Markers					
NM_002006	Fibroblast growth factor 2 (basic)	FGF2 (bFGF)	0.7953	0.6199	
NM_000207	Insulin	INS	1.5621	0.1746	
NM_002309	Leukemia inhibitory factor	LIF	0.8646	0.8617	
NM_002701	Octamer-binding transcription factor 4	OCT4	1.4192	0.4964	
NM_003106	SRY (sex determining region Y)-box 2	SOX2	0.8048	0.6364	
NM_198253	Telomerase reverse transcriptase	TERT	0.8910	0.7668	
NM_033131	Wingless-type MMTV integration site family, member 3A	WNT3A	0.6201	0.1349	
NM_174900	Zinc finger protein 42 homolog (mouse)	ZFP42	0.8237	0.4309	
MSC-Specific Markers					
NM_001627	Activated leukocyte cell adhesion molecule	ALCAM	1.3080	0.8221	
NM_001150	Alanyl (membrane) aminopeptidase	ANPEP	0.7301	0.0570	
NM_004346	Caspase 3	CASP3	0.9429	0.6789	
NM_000610	CD44 molecule (Indian blood group)	CD44	0.7366	0.1873	
NM_000118	Endoglin	ENG	0.8326	0.1912	
NM_004448	V-erb-b2 erythroblastic leukemia viral oncogene homolog 2, neuro/glioblastoma derived oncogene homolog (avian)	ERBB2 (HER2)	1.4973	0.2182	
NM_002033	Fucosyltransferase 4	FUT4	2.7344	0.1352	
NM_003508	Frizzled family receptor 9	FZD9	1.0164	0.9788	
NM_000210	Integrin, alpha 6	ITGA6	0.7706	0.0587	
NM_002210	Integrin, alpha V	ITGAV	0.6457	0.1285	
NM_006500	Melanoma cell adhesion molecule	MCAM	2.3341	0.2525	
NM_002507	Nerve growth factor receptor	NGFR	2.1012	0.6663	
NM_002526	5'-nucleotidase, ecto (CD73)	NT5E	0.7620	0.4739	
NM_002609	Platelet-derived growth factor receptor, beta polypeptide	PDGFRB	2.1955	0.1926	
NM_006017	Prominin 1	PROM1	0.8566	0.9428	
NM_006288	Thy-1 cell surface antigen	THY1	1.4852	0.8937	
NM_001078	Vascular cell adhesion molecule 1	VCAM1	1.4291	0.3592	
Other Genes Associated with MSC					
NM_001154	Annexin A5	ANXA5	0.7301	0.0570	
NM_001709	Brain-derived neurotrophic factor	BDNF	0.6672	0.1248	
NM_199173	Bone gamma-carboxyglutamate (gla) protein	BGLAP (Osteocalcin)	0.8570	0.3911	
NM_001719	Bone morphogenetic protein 7	BMP7	1.0223	0.8279	
NM_000088	Collagen, type I, alpha 1	COLA1	0.9038	0.6852	
NM_000758	Colony stimulating factor 2 (granulocyte-macrophage)	CSF2	0.8835	0.7697	
NM_000759	Colony stimulating factor 3 (granulocyte)	CSF3	0.5650	0.4167	
NM_001904	Catenin (cadherin-associated protein), beta 1, 88kDa	CTNNB1	0.8108	0.1780	
NM_001963	Epidermal growth factor	EGF	1.1041	0.3717	
NM_000148	Fucosyltransferase 1 (galactoside 2-alpha-L-fucosyltransferase, H blood group)	FUT1	1.0851	0.9881	
NM_002097	General transcription factor IIIA	GTF3A	0.7706	0.0587	
NM_000601	Hepatocyte growth factor (hepapoietin A; scatter factor)	HGF	1.0719	0.7792	
NM_000201	Intercellular adhesion molecule 1	ICAM1	0.9367	0.2667	
NM_000619	Interferon, gamma	IFNG	0.8852	0.6453	
NM_000618	Insulin-like growth factor 1 (somatomedin C)	IGF1	0.6575	0.4696	
NM_000572	Interleukin 10	IL10	0.8646	0.7706	
NM_000576	Interleukin 1, beta	IL1B	1.1277	0.6064	
NM_000600	Interleukin 6 (interferon, beta 2)	IL6	0.5735	0.3332	
NM_002211	Integrin, beta 1	ITGB1	0.8252	0.1995	
NM_003994	KIT ligand	KITLG	1.3767	0.4027	
NM_004530	Matrix metallopeptidase 2	MMP2	0.8092	0.9990	
NM_006617	Nestin	NES	2.8428	0.1399	
NM_007083	Nudix (nucleoside diphosphate linked moiety X)-type motif 6	NUDT6	0.6588	0.0966	
NM_033198	Phosphatidylinositol glycan anchor biosynthesis, class S	PIGS	0.7380	0.0112	*
NM_002838	Protein tyrosine phosphatase, receptor type, C	PTPRC	0.9385	0.4442	
NM_012434	Solute carrier family 17 (anion/sugar transporter), member 5	SLC17A5	0.7650	0.2122	
NM_003239	Transforming growth factor, beta 3	TGFB3	2.8015	0.0747	
NM_000594	Tumor necrosis factor alpha	TNFA	1.3130	0.9124	
NM_003380	Vimentin	VIM	1.0246	0.6979	
NM_000552	Von Willebrand factor	VWF	0.7469	0.9309	
MSC Differentiation Markers					
NM_000927	ATP-binding cassette, sub-family B, member 1	ABCB1	0.6870	0.2662	
NM_001613	Actin, alpha 2, smooth muscle, aorta	ACTA2	1.1200	0.3465	
NM_001200	Bone morphogenetic protein 2	BMP2	0.7372	0.3316	
NM_130851	Bone morphogenetic protein 4	BMP4	0.4244	0.0318	*
NM_001718	Bone morphogenetic protein 6	BMP6	0.5956	0.2765	
NM_004465	Fibroblast growth factor 10	FGF10	0.8527	0.8111	
NM_000557	Growth differentiation factor 5	GDF5	1.1011	0.7284	
NM_001001557	Growth differentiation factor 6	GDF6	0.8882	0.3208	
NM_182828	Growth differentiation factor 7	GDF7	1.5621	0.1746	
NM_004864	Growth differentiation factor 15	GDF15	1.5164	0.4034	
NM_003642	Histone acetyltransferase 1	HAT1	0.6457	0.1285	
NM_004964	Histone deacetylase 1	HDAC1	1.0645	0.6023	
NM_000545	HNF1 homeobox A	HNF1A	1.3150	0.2274	
NM_000887	Integrin, alpha X (complement component 3 receptor 4 subunit)	ITGAX	1.3150	0.2274	
NM_000214	Jagged 1	JAG1	0.9097	0.9708	
NM_002253	Kinase insert domain receptor (a type III receptor tyrosine kinase)	KDR	0.7721	0.4877	
NM_017617	Notch 1	NOTCH1	1.2610	0.6630	
NM_003884	KAT2B K acetyltransferase 2B (p300/CBP-associated factor)	KAT2B (PCAF)	0.7324	0.0187	*
NM_015869	Peroxisome proliferator-activated receptor gamma	PPARG	2.8549	0.2081	
NM_005607	PTK2 protein tyrosine kinase 2	PTK2	0.9267	0.9863	
NM_001664	Ras homolog gene family, member A	RHOA	0.7527	0.0553	
NM_004348	Runt-related transcription factor 2	RUNX2	0.8218	0.8104	
NM_005359	SMAD family member 4	SMAD4	0.9616	0.8781	
NM_020429	SMAD specific E3 ubiquitin protein ligase 1	SMURF1	1.2223	0.2759	
NM_022739	SMAD specific E3 ubiquitin protein ligase 2	SMURF2	0.6907	0.0917	
NM_000346	SRY (sex determining region Y)-box 9	SOX9	0.6700	0.8982	
NM_181486	T-box 5	TBX5	2.3522	0.4270	
NM_000660	Transforming growth factor, beta 1	TGFB1	1.1680	0.4280	
House Keeping Gene					
NM_001101	Actin, beta	ACTB	1.0199	0.7951	
NM_004048	Beta-2-microglobulin	B2M	1.0868	0.5985	
NM_002046	Glyceraldehyde-3-phosphate dehydrogenase	GAPDH	1.2408	0.1401	
NM_000194	Hypoxanthine phosphoribosyltransferase 1	HPRT1	0.7455	0.0567	
NM_001002	Ribosomal protein, large, P0	RPLP0	0.9754	0.7501	

*p<0.05 between HC-MSCs (n = 6) and KD-MSCs (n = 9).

### Quantitative RT-PCR Analysis of PCAF Expression

Total RNA was extracted using an RNeasy Mini Kit, and cDNA was synthesized using a RT2 First Strand Kit (Qiagen). An RT2 qPCR Primer Assay (Cat. No: PPH02176F; Qiagen) was used to analyze PCAF expression. PCR was performed using the ABI 7300 Real-time PCR System and RT2 SYBER Green Master Mix. All samples were tested in duplicate. Dissociation curves were analyzed after each reaction to assess quantification specificity. All samples were normalized to β-actin expression using the relative standard curve method.

### Western Blot Analysis

Protein samples for western blot analysis were prepared as described previously [22]. Briefly, MSCs (passages 3–5) were washed three times with ice-cold PBS and then treated with lysis buffer (50 mM Tris-HCl, pH 7.5, containing 2% SDS (Sigma-Aldrich) and a protease inhibitor cocktail (Roche, Mannheim, Germany). Samples were centrifuged for 1 h at 18,000× *g* at 4°C. The supernatants were collected as whole cell lysates. Protein concentrations were estimated using a DC protein assay (Bio-Rad) with a bovine serum albumin standard. Equal amounts of proteins (10 µg) were resolved by SDS-polyacrylamide gel electrophoresis on 4–20% acrylamide gradient gels (Bio-Rad) and then transferred onto a polyvinylidene fluoride microporous membrane (Millipore, Billerica, MA). The membranes were blocked with a blocking reagent (Toyobo, Tokyo, Japan) and then incubated with each primary antibody. The primary antibodies used were: rabbit anti-PCAF, rabbit anti-HIF-1α (Cell Signaling Technology), rabbit anti-VEGF (Santa Cruz Biotechnology, Santa Cruz, CA) and rabbit anti-β-actin (Cell Signaling Technology). After washing, the membranes were incubated with a peroxidase-labeled secondary antibody (Nichirei, Tokyo, Japan) and visualized using Immunostar LD (Wako). Images were captured digitally using a ChemiDoc XRS+ (Bio-Rad) and analyzed by Image Lab 2.0.1 software (Bio-Rad).

### PCAF, HIF-1α, and VEGF Expression under Hypoxia

Because PCAF acetylates HIF-1α under hypoxic conditions and modulates the activity and protein stability of HIF-1α [22], we investigated the effect of hypoxia on HIF-1α expression in HC-MSCs and KD-MSCs. Quantitative RT-PCR and western blotting were used to analyze PCAF, HIF-1α, and VEGF expression in HC-MSCs and KD-MSCs at 24 h under normoxia and hypoxia (1% O_2_).

### Statistical Analysis

Experiments were performed using independently isolated MSCs from all 15 participants. All data are presented as means ± SE. Data were analyzed using the (two-tailed) paired *t*-test or unpaired *t*-test. Statistical significance was defined as *P*<0.05. Experimental data were analyzed using GraphPad Prism version 5.0 software (Graphpad Software, San Diego, CA) and Microsoft Excel (Microsoft, Redmond, WA).

### Directed *In Vivo* Angiogenesis Assay

A directed *in vivo* angiogenesis assay (DIVAA; Trevigen, Gaithersburg, MD) was performed according to the manufacturer's protocol. Briefly, implant-grade silicone cylinders closed at one end (angioreactors) were filled with 18 µl Trevigen's basement membrane extract (Trevigen) with 37.5 ng VEGF and 12.5 ng basic fibroblast growth factor (bFGF; positive control, *n* = 8), PBS (negative control, *n* = 8), or 1×10^6^ HC/KD-MSCs in serum-free αMEM (*n* = 8). MSCs were selected from one cell line each of HC-MSCs and KD-MSCs at passage 3. The angioreactors were implanted subcutaneously into 8-week-old nude mice (Sankyo Laboratory Service, Tokyo, Japan). At 9 days after implantation, the mice were sacrificed and the angioreactors were removed, photographed, and stained with FITC-labeled lectin as an endothelial cell-selective reagent [23] to quantify the invasion of endothelial cells into the angioreactors [24]. Fluorescence was measured in 96-well black plates (Thermo Fisher Scientific, Roskilde, Denmark) using an ARVO MX model spectrofluorometer (485 nm excitation and 510 nm emission; Perkin Elmer, Boston, MA). The mean relative fluorescence ± S.E. were determined for triplicate assays. Statistical analysis (Unpaired t-test) was performed using GraphPad Prism version 5.0 software (Graphpad Software, San Diego, CA).

## Results

### MSC Isolation

MSCs were successfully isolated from all six healthy controls (HC-MSCs) and nine ESKD patients (KD-MSCs). MSC donor characteristics are depicted in Table 1. All ESKD patients had received standard dialysis therapy for renal insufficiency. Additionally used medications are listed in Table 1.

### Characterization of MSCs

**Figure 1:**
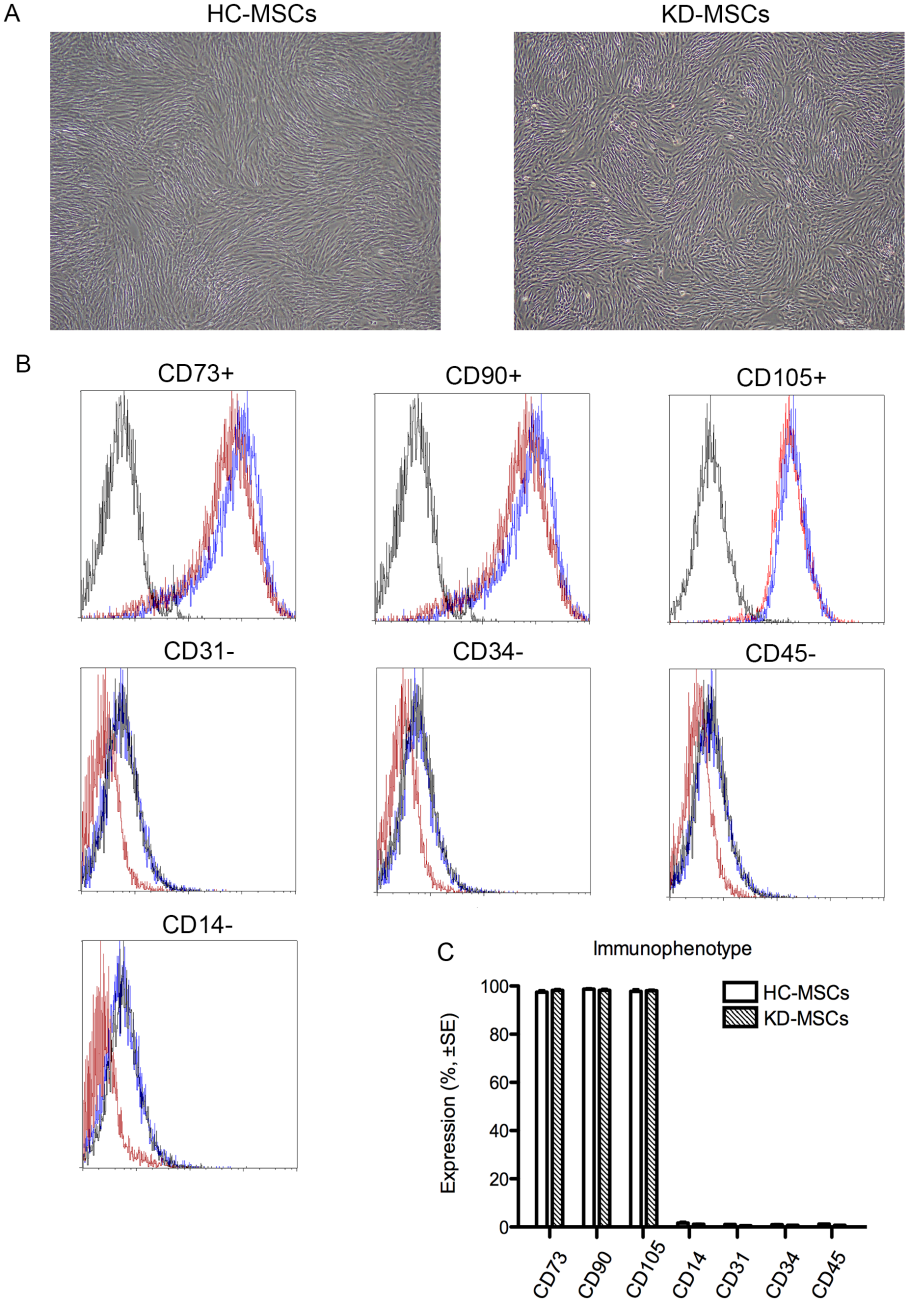
Characteristics of mesenchymal stem cells from healthy controls (HC-MSCs) and patients with ESKD (KD-MSCs). (A) Representative images of HC-MSCs (left) and KD-MSCs (right; original magnification, ×100). (B) Flow cytometric analysis of cell surface marker expression of HC-MSCs (solid lines; *n* = 6) and KD-MSCs (dashed lines; *n* = 9). Isotype-matched IgG controls are represented by solid histograms. (C) Comparison of cell surface marker expression in HC-MSCs (*n* = 6) and KD-MSCs (*n* = 9). The percentages of positive cells are shown. Data are the mean ± SE. There were no significant differences.

HC-MSCs and KD-MSCs cultured in standard culture medium showed a similar spindle-shaped morphology (Figure 1A). Surface markers of all the established MSC lines were characterized by flow cytometric analysis. Both HC-MSCs and KD-MSCs were positive for CD73, CD90, and CD105, and negative for CD14, CD31, CD34, and CD45. MSC surface markers CD73, CD105, and CD90 were expressed in >95% of cell populations (Figure 1B), and CD14, CD31, CD34, and CD45 were expressed in <2% of cell populations (Figure 1B). No differences were found in the immunophenotypes of cultured HC-MSCs and KD-MSCs (Figure 1C).

### Adipogenic, Osteogenic, and Chondrogenic Differentiation

**Figure 2:**
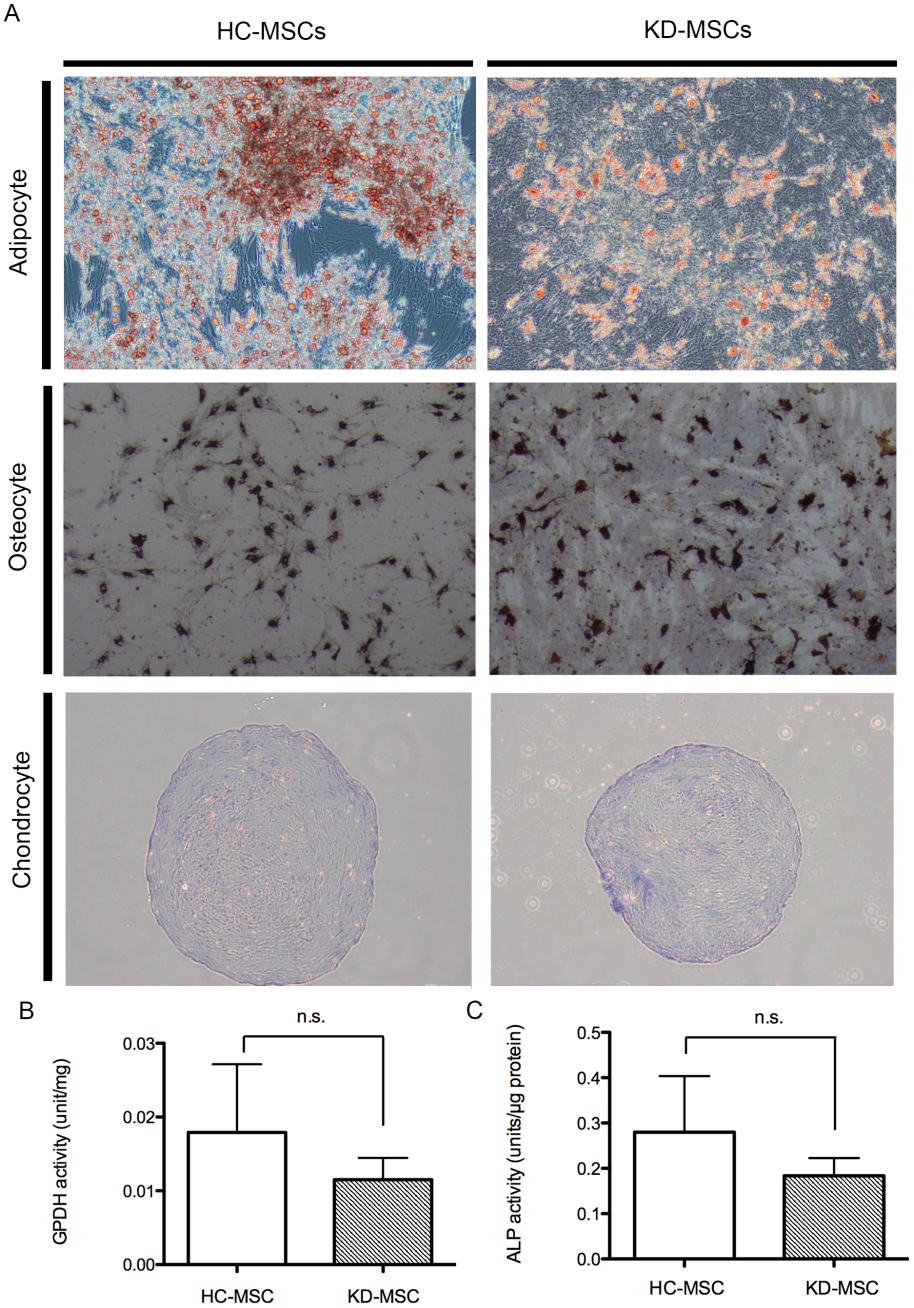
Differentiation capacities of HC-MSCs and KD-MSCs. (A) Adipogenic differentiation of HC-MSCs (top and left) and KD-MSCs (top and right) was examined after 2 weeks of culture under adipogenic conditions by Sudan III staining (original magnification, ×100). Osteogenic differentiation of HC-MSCs (second from top and left) and KD-MSCs (second from top and right) was examined after 4 weeks of culture under osteogenic conditions by von Kossa staining (original magnification, ×100). Chondrogenic differentiation of HC-MSCs (bottom and left) and KD-MSCs (bottom and right) was examined after 3 weeks of culture under chondrogenic conditions by Safranin O/Fast green staining (original magnification, ×100). (B) GPDH activity of cells was measured to compare the adipogenic differentiation capacities of HC-MSCs (*n* = 5) and KD-MSCs (*n* = 5). Data are expressed as the mean ± standard error (SE). ^*^ *P*<0.05. (C) ALP activity of the cells was measured to indicate their osteogenic differentiation capacity (*n* = 4). Data are expressed as the mean ± SE. ^*^ *P*<0.05.

Histopathological examination by Sudan III, von Kossa, Safranin O, and Fast green staining was performed to identify adipogenic, osteogenic, and chondrogenic lineages, respectively (Figure 2A). We also found no significant differences in GPDH (*n* = 5 for KD-MSCs and HC-MSCs) or ALP (*n* = 5 for KD-MSCs and HC-MSCs) activities, representing adipogenic and osteogenic differentiation, respectively, in HC-MSCs and KD-MSCs (Figure 2B and 2C).

### MSC Senescence and Proliferation

**Figure 3:**
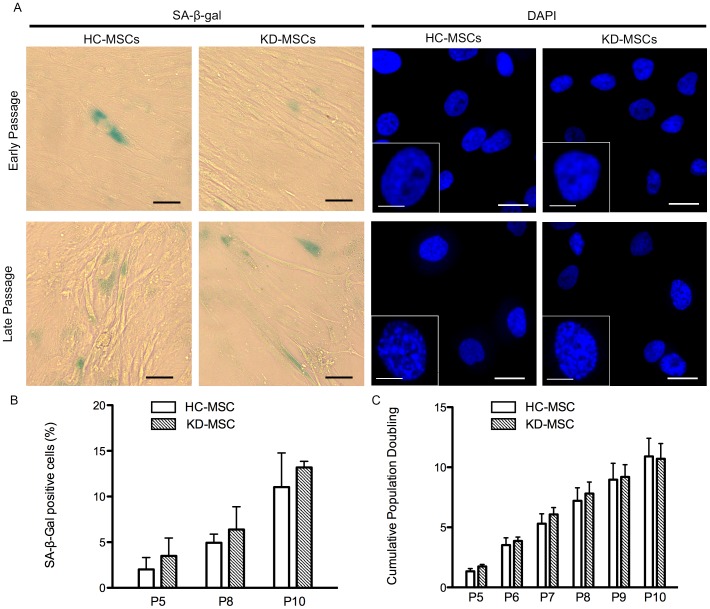
Proliferation and senescence of HC-MSCs and KD-MSCs. (A) Representative images of HC-MSCs and KD-MSCs (magnification, ×40). Left columns show assessment of senescence using the senescence biomarker SA-β-gal (green) in HC-MSCs and KD-MSCs. Black scale bars represent 50 µm. Right columns show DAPI staining of senescence-associated heterochromatic foci (SAHF) in MSC DNA foci. White scale bars represent 10 µm. Insets show an enlargement of DAPI staining (white scale bars represent 5 µm). Early passage: P5; late passage: P10. (B) Quantitative assessment of SA-β-gal positive cells. Data are the mean ± SE (*n* = 4). ^*^ *P*<0.05. (C) Cumulative population doublings (PDs) of HC-MSCs (*n* = 5) and KD-MSCs (*n* = 5) from passage 5–10. Data are expressed as the mean ± SE. ^*^ *P*<0.05. Experiments were performed in triplicate.

MSCs possess a limited lifespan during *in vitro* culture because they undergo senescence [19], characterized by cell cycle arrest, telomere shortening, and altered morphology. To assess the percentage of cells undergoing senescence, we used SA-β-gal as a senescence marker. The percentages of β-galactosidase-positive cells increased from passage 5 to 10, but there was no significant difference in β-galactosidase positivity in HC-MSCs (*n* = 4) and KD-MSCs (*n* = 4) at passages 5, 8, and 10 (Figure 3A and 3B). These results suggest that there was no significant difference in the proliferation or senescence of HC-MSCs and KD-MSCs.

A second senescence assay determined the formation of SAHF, which are visible as microscopically discernible, punctate DNA foci in DAPI-stained senescent cells [25]. As shown in Figure 3A (right columns), late passage (P10) MSCs showed punctuated DNA foci, while early passage (P5) MSCs displayed several small nucleoli and a more uniform DAPI staining pattern. We observed a similar formation of SAHF between HC-MSCs and KD-MSCs in early and late passages.

The proliferation potentials of HC-MSCs and KD-MSCs were evaluated over six passages and PD levels were measured from passage 5–10. HC-MSCs and KD-MSCs displayed similar cumulative PDs with a peak of 10.89±1.52 and 10.71±1.26, respectively, at passage 10 (*P*<0.05 versus controls) (Figure 3C). Cell proliferation rates of HC-MSCs (*n* = 5) and KD-MSCs (*n* = 5) showed no significant difference.

### RT-PCR Array Analysis of MSCs

**Figure 4:**
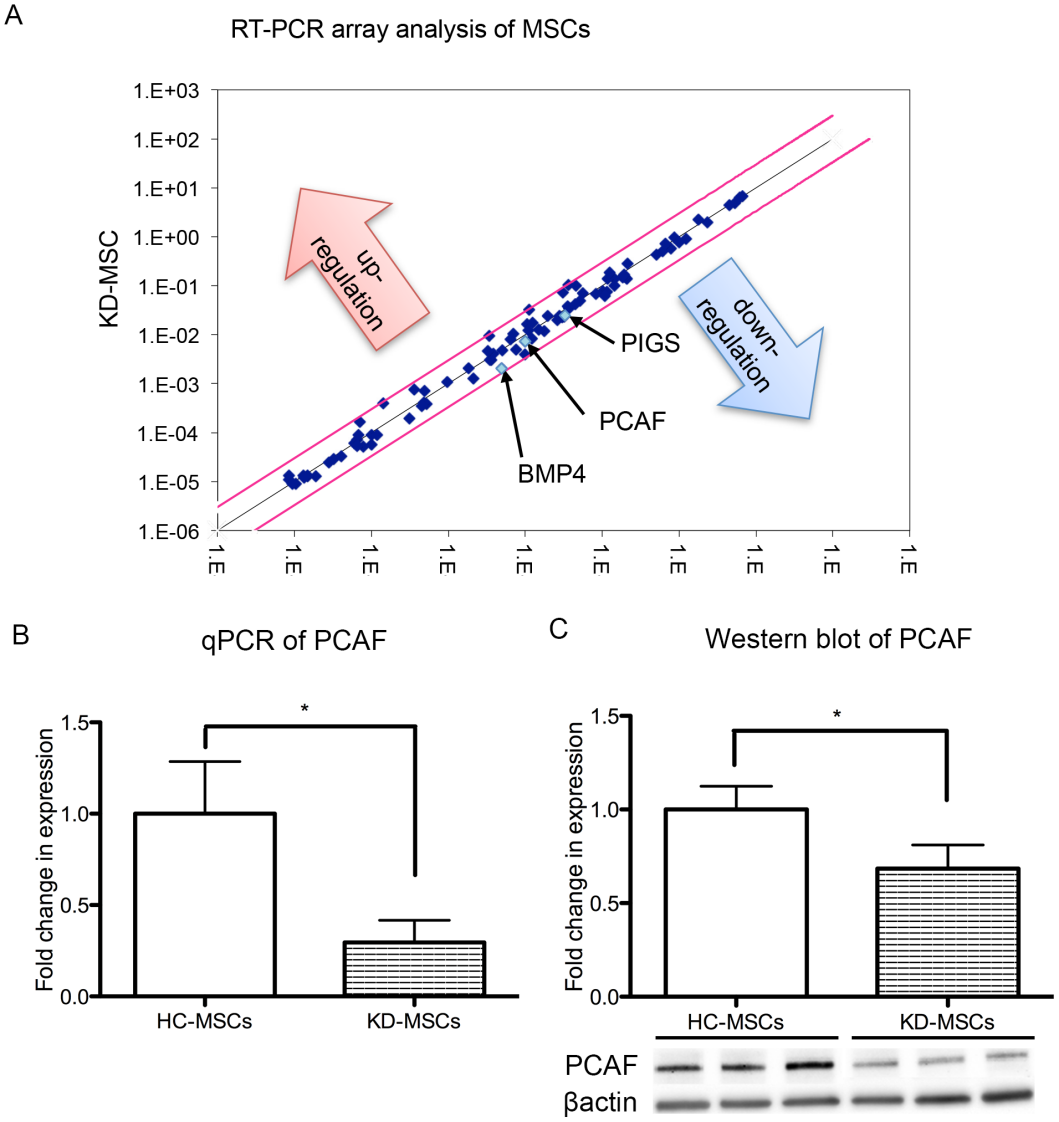
Real-time PCR array, quantitative PCR, and western blot analyses of MSCs. (A) Downregulation of multiple stem cell-relevant transcription factors in KD-MSCs (*n* = 9) compared with HC-MSCs (*n* = 6). The fold change [2∧(−ΔΔCt)] is the normalized gene expression [2∧(−ΔCt)] in KD-MSCs relative to that in HC-MSCs. *P*-values were calculated based on the Student's *t*-test of replicate 2∧(−ΔCt) values for each gene in HC-MSCs and KD-MSCs. *P*<0.05 is indicated with black arrows. (B) Quantitative PCR was performed to measure the levels of gene expression in HC-MSCs (*n* = 6) and KD-MSCs (*n* = 6). Data are expressed as the mean ± SE. ^*^ *P*<0.05. (C) Western blot analysis of PCAF in KD-MSCs and HC-MSCs. PCAF expression was decreased in KD-MSCs (*n* = 9) compared with HC-MSCs (*n* = 6). PCAF protein levels are expressed relative to β-actin. Data are expressed as the mean ± SE. ^*^ *P*<0.05.

Quantitative RT-PCR array analysis profiled the expression of 84 key genes, including those involved in stemness and self-renewal of MSCs (Table 2), and revealed distinct expression patterns in HC-MSCs (*n* = 6) and KD-MSCs (*n* = 9). Compared with HC-MSCs, we found significantly lower expression of *PCAF*, *BMP4*, and *PIGS* (fold differences: 0.73, 0.42, and 0.72, respectively) in KD-MSCs (Figure 4A, Table 2), suggesting a functional difference in HC-MSCs and KD-MSCs. There were no significant differences in expression of the other 81 genes (Table 2).

### Quantitative RT-PCR and Western Blot Analyses of MSCs

Quantitative RT-PCR was performed to verify the downregulation of *PCAF* expression in KD-MSCs, and confirmed that *PCAF* expression levels were significantly downregulated in KD-MSCs (*n* = 6, ^*^ *P*<0.05, Figure 4B). Similarly, PCAF protein expression was significantly decreased in KD-MSCs (*n* = 9) compared with HC-MSCs (*n* = 6) as shown by western blotting (^*^ *P*<0.05, Figure 4C).

### Downregulation of PCAF, HIF-1α, and VEGF Gene and Protein Expression in KD-MSCs Cultured under Hypoxic Conditions

**Figure 5:**
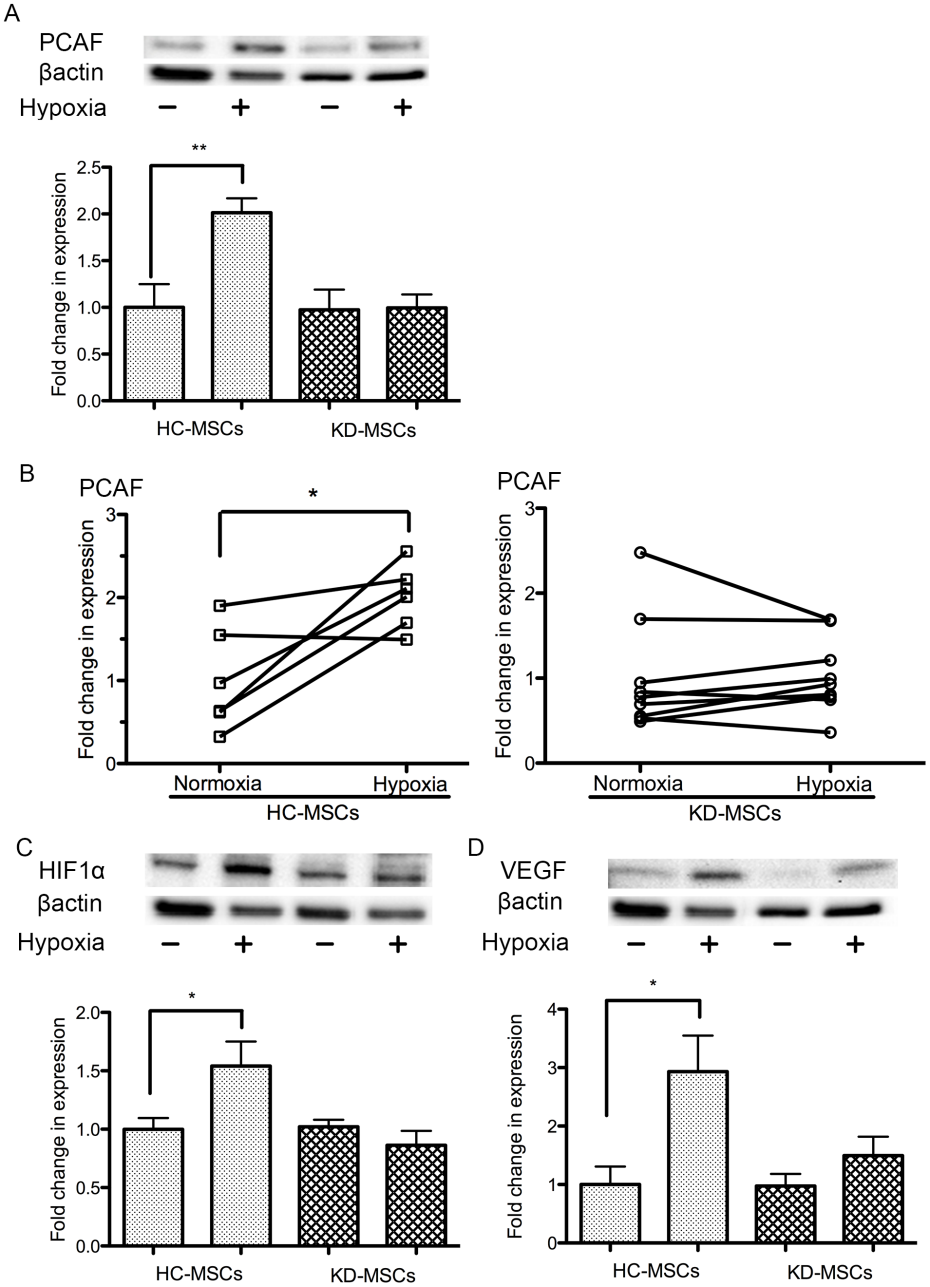
Western blot analysis of PCAF, HIF-1α, and VEGF expression under hypoxia and normoxia. (A) Western blot analysis of PCAF expression in KD-MSCs (*n* = 9) and HC-MSCs (*n* = 6) under normoxia and hypoxia (1% O_2_). Data are the mean ± SE. ^**^P<0.01 normoxia versus hypoxia in HC-MSCs (two-tailed, unpaired *t*-test). (B) Western blot analysis of PCAF expression at 24 h under hypoxia showed it to be clearly upregulated in HC-MSCs. There was no change in PCAF in KD-MSCs under hypoxia. Data are the mean ± SE (HC-MSCs *n* = 6, KD-MSCs *n* = 9; ^*^ *P*<0.05 versus normoxia, two-tailed, paired *t*-test). (C) Western blot analysis of HIF-1α expression in KD-MSCs (*n* = 9) and HC-MSCs (*n* = 6) under normoxia and hypoxia. Data are the mean ± SE. ^*^ *P*<0.05 versus normoxia (two-tailed, unpaired *t*-test). (D) Western blot analysis of VEGF expression in KD-MSCs (*n* = 9) and HC-MSCs (*n* = 6) under normoxia and hypoxia. Data are the mean ± SE. ^*^ *P*<0.05 versus normoxia (two-tailed, unpaired *t*-test). (A–D) MSC lines were isolated independently.

We hypothesized that PCAF in human MSCs would be upregulated under hypoxia, so investigated the gene and protein expression of PCAF, HIF-1α, and the downstream factor VEGF in HC-MSCs and KD-MSCs under normoxia (21% O_2_) and hypoxia (1% O_2_) at 24 h. PCAF protein expression was significantly upregulated under hypoxia (1% O_2_, 24 h) in HC-MSCs (*n* = 6, *P*<0.05, Figure 5A, 5B), but this was not observed in KD-MSCs (*n* = 9, *P*<0.05, Figure 5A, 5B). Similarly, HIF-1α expression was significantly upregulated under hypoxia in HC-MSCs (*n* = 6, *P*<0.05, Figure 5C), but not in KD-MSCs (*n* = 9, *P*<0.05, Figure 5C). Furthermore, the enhancement of VEGF expression under hypoxic conditions, which is regulated by HIF-1α [26], was significantly decreased in KD-MSCs under hypoxia (*n* = 9, *P*<0.05 Figure 5D). We demonstrated that KD-MSCs did not upregulate the protein expression of PCAF, HIF-1α, or VEGF under hypoxia, suggesting that the hypoxic response might be blunted in KD-MSCs.

### DIVAA of MSCs

**Figure 6:**
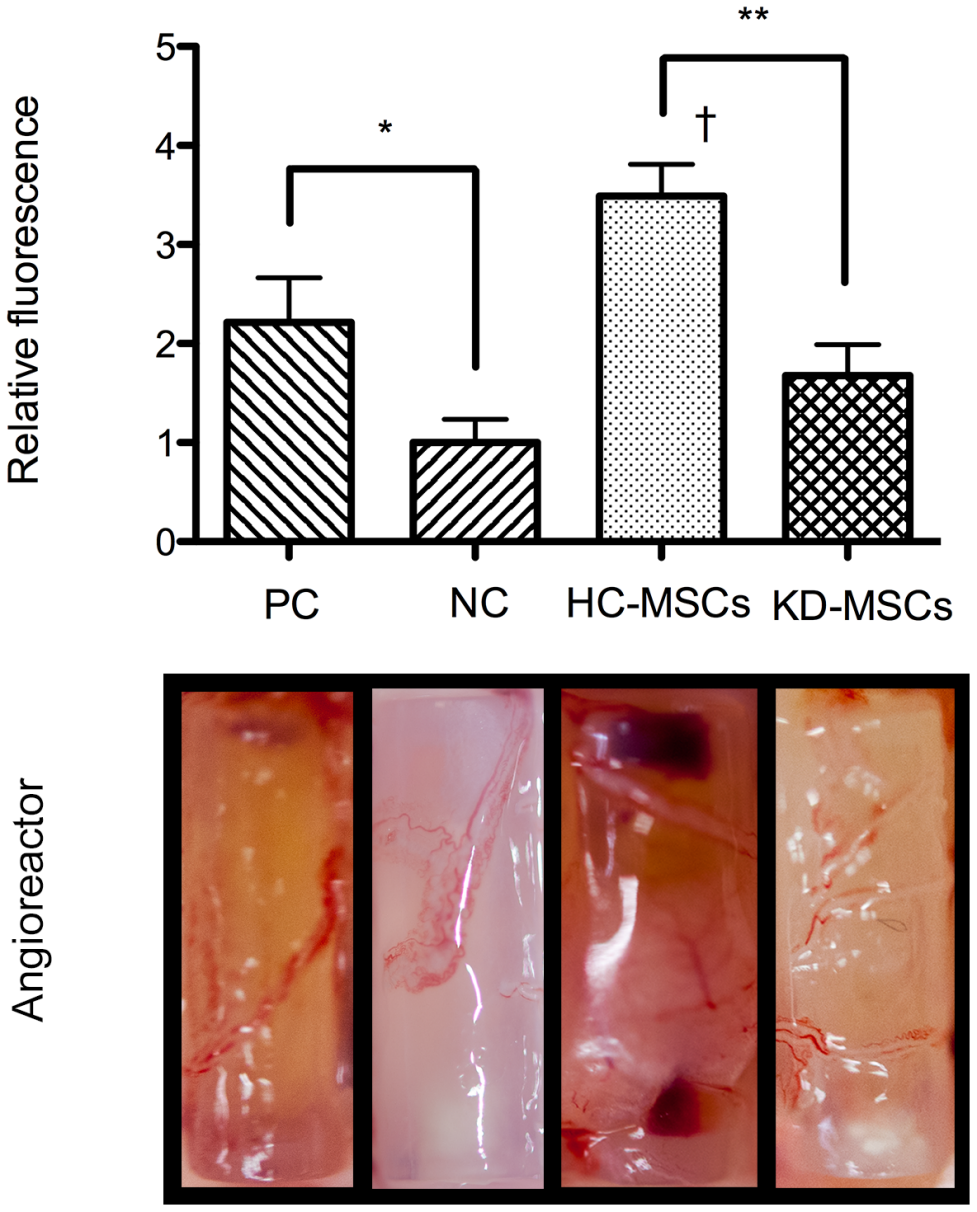
Directed *in vivo* angiogenesis assay. Angioreactors containing HC-MSCs (*n* = 8), KD-MSCs (*n* = 8), 37.5 ng VEGF and 12.5 ng bFGF (positive control, *n* = 8), or PBS (negative control, *n* = 8) were used in a DIVAA. The photographs show blood vessel growth into the angioreactors. The bar graph shows the average ± S.E. of triplicates. ^**^ *P*<0.01, HC-MSCs versus KD-MSCs; ^*^ *P*<0.05, PC versus NC; ^†^ *P*<0.05, HC-MSCs versus PC. PC: Positive control; NC: Negative control.

Because a previous study has proposed that PCAF is a key regulator of angiogenesis [27], we tested angiogenesis activation of HC-MSCs and KD-MSCs *in vivo*. A DIVAA assesses angiogenesis activation, which provides quantitative and reproducible results [28]. The results showed significant blood vessel growth into angioreactors containing HC-MSCs, which was similar to that seen in positive controls (VEGF/bFGF). However, we observed only slight growth into angioreactors containing KD-MSCs (Fig. 6, lower panel). Thus, HC-MSCs showed better angiogenesis activation than that of KD-MSCs.

Furthermore, measurement of FITC-lectin bound to the endothelial contents of angioreactors supported these findings and demonstrated reduced fluorescence in angioreactors containing KD-MSCs compared with the strong fluorescence seen in angioreactors containing HC-MSCs (Fig. 6, bar graph). These results support the notion that KD-MSCs with low expression of PCAF exhibit lower angiogenesis activation than that of HC-MSCs.

## Discussion

Stem cells from patients with ESKD are needed for kidney regeneration, but it is not yet known if stem cells exposed to long-term uremic conditions will function normally. To explore whether the acquired disease environment causes long-term changes and influences the cell environment, we compared MSCs from ESKD patients and those with normal kidney function by RT-PCR array. After culturing KD-MSCs under normal conditions for 2–3 weeks, *PCAF*, *PIGS*, and *BMP4* expression was shown to differ significantly between HC-MSCs and KD-MSCs.

PCAF plays a role in the regulation of differentiation, angiogenesis, cell cycle progression, and gluconeogenesis [27], [29], [30], and is one of the many factors involved in epigenetics [31]. However, the mechanisms behind its actions have not yet been elucidated, particularly its role in angiogenesis. A previous study of PCAF^−/−^ mice indicated that PCAF acts as a master switch for effective arteriogenesis [27], while another report demonstrated a role for PCAF in angiogenic tubule formation because human umbilical vein endothelial cells transfected with PCAF siRNA showed a significant reduction of angiogenic tubule formation [32].

A previous study used proteomics to show that PCAF was upregulated under hypoxia in the rat kidney fibroblast NRK-49F cell line [33]. Additionally, PCAF acetylates HIF-1α under hypoxic conditions, which fine tunes its transcriptional activity, increases its protein stability, and causes modulation of cellular responses [20], [33]–[35]. HIF-1α is known to be a key regulator of angiogenesis and controls the expression of multiple angiogenic factors including VEGF [26], [36].

Interestingly, we found that PCAF expression under hypoxia was increased in HC-MSCs (Figure 5A and 5B), but not KD-MSCs in the present study. Previously, studies in other animal cell types have suggested that PCAF is upregulated under hypoxia [37], but no investigations have been carried out into the hypoxic response and the influence of long-term uremic conditions on PCAF in human MSCs. Because PCAF is a facilitator for HIF-1α and VEGF [29], [38], we investigated the hypoxic responses of HIF-1α and VEGF by western blotting and detected low levels of HIF-1α and VEGF protein expression in KD-MSCs under hypoxic conditions (Figure 5C and 5D). Consequently, it appears that the hypoxic response is significantly blunted in KD-MSCs with low PCAF expression, but not in HC-MSCs.

A previous report found that uremic conditions decreased HIF-1α and VEGF expression under hypoxia in mice [13]. However, this study did not refer to PCAF, and it was thought that Akt phosphorylation might underlie abnormal cell survival and the angiogenic functions of MSCs in uremia. Uremia is an illness accompanying kidney failure that cannot be explained by alteration of the extracellular volume, inorganic ion concentrations, or lack of known renal synthetic products [39]. The precise mechanisms have yet to be identified for uremia and hypoxia, but the finding that KD-MSCs show downregulation of PCAF expression, poor angiogenesis activation *in vivo*, and blunting of the hypoxic response might be helpful in their elucidation in ESKD patients.

Kidney development is associated with coordinated branching of the renal tubular and vascular systems, and hypoxia has been proposed as a major regulatory factor in this process [40]. We have developed a regeneration method involving transplantation of a metanephros into the omentum to attract the host veins into the graft and generate urine in hypoxic tissues [7]–[9]. Because angiogenesis and hypoxia might play a role in kidney regeneration, it is important to investigate the hypoxic responses of PCAF, HIF-1α, and VEGF in KD-MSCs and HC-MSCs. Furthermore, PCAF is a member of the histone acetyltransferases (HATs). HATs promote an open chromatin configuration and transcriptional activation. Epigenetic modifications by HATs and histone deacetylases (HDACs) have a direct effect on gene regulation, cell differentiation, and cellular stability during renal development [41]. In fact, even though the role of HDACs is well studied [42], little is known about the role of HATs during metanephric kidney development [41].

There are currently no available assays to directly test for PCAF activity, so we cannot exclude histone acetyltransferase activity as an underlying mechanism for the effect of PCAF. Our data suggest that, within the context of the hypoxic response, it is more likely that PCAF functions as a transcriptional coactivator to regulate HIF-1α expression. The involvement of global gene expression and epigenetic effects is also unclear and should be investigated further. While it would be useful to demonstrate the regeneration of kidney tissue from KD-MSCs using our previous methods [7]–[9], and directly compare the differences between KD-MSCs and HC-MSCs, this technique is very difficult. The cell transplantation efficiency has a 3% success rate and only about 30% of transplantations generate a neo-kidney. Thus, we did not apply this method in the current study because of its very low success rate.

In conclusion, we demonstrated differences in the gene and protein expression of MSCs from ESKD patients and healthy individuals using a PCR array and western blot analysis. We found that long-term uremic conditions led to persistent and systematic downregulation of *in vitro* gene and protein expression of PCAF and poor *in vivo* angiogenesis activation of MSCs from patients with ESKD. Furthermore, we demonstrated that the hypoxic responses of PCAF, HIF-1α, and VEGF were significantly blunted in MSCs from ESKD patients. We propose that the transcriptional regulation by low levels of PCAF might be inappropriately controlled by environmental factors representing long-term ESKD. Low expression of PCAF induced by long-term ESKD may lead to downregulation of HIF-1α and VEGF in KD-MSCs under hypoxia. These findings should help to elucidate the mechanisms of the effects of uremic toxins. Further studies are needed to clarify the relationship of CKD and the downregulation of PCAF. Moreover, based on our study, the role of PCAF may be investigated further in epigenetic mechanisms during kidney development.

## References

[1] The Japanese Society of Nephrology (2012) Clinical practice guidebook for diagnosis and treatment of chronic kidney disease. Jpn J Nephrol 54: 1031–1189. 23387281

[2] Berger A, Edelsberg J, Inglese GW, Bhatttacharyya SK, Oster G (2009) Cost comparison of peritoneal dialysis versus hemodialysis in end-stage renal disease. Am J Manag Care 15: 509–518. 19670954

[3] Wolfe RA, Ashby VB, Milford EL, Ojo AO, Ettenger RE, et al (1999) Comparison of mortality in all patients on dialysis, patients on dialysis awaiting transplantation, and recipients of a fiomp cadaveric transplant. N Engl J Med 341: 1725–1730. 10580071 10.1056/NEJM199912023412303

[4] Sonoda T, Takahara S, Takahashi K, Uchida K, Ohshima S, et al (2003) Outcome of 3 years of immunosuppression with tacrolimus in more than 1,000 renal transplant recipients in Japan. Transplantation 75: 1999–024. 10.1097/01.TP.0000040867.67360.9F 12548123

[5] Badylak SF, Taylor D, Uygun K (2011) Whole-organ tissue engineering: decellularization and recellularization of three-dimensional matrix scaffolds. Annu Rev Biomed Eng 15: 27–53. 10.1146/annurev-bioeng-071910-124743 PMC10887492 21417722

[6] Xinaris C, Benedetti V, Rizzo P, Abbate M, Corna D, et al (2012) In vivo maturation of functional renal organoids formed from embryonic cell suspensions. J Am Soc Nephrol 23: 1857–1868. 23085631 10.1681/ASN.2012050505 PMC3482737

[7] Yokoo T, Ohashi T, Shen JS, Sakurai K, Miyazaki Y, et al (2005) Human mesenchymal stem cells in rodent whole embryo culture are reprogrammed to contribute kidney tissues. Proc Natl Acad Sci U S A 102: 3296–3300. 15728383 10.1073/pnas.0406878102 PMC552897

[8] Yokoo T, Fukui A, Ohashi T, Miyazaki Y, Utsunomiya Y, et al (2006) Xenobiotic kidney organogenesis from human mesenchymal stem cells using a growing rodent embryo. J Am Soc Nephrol 17: 1026–1034. 16524947 10.1681/ASN.2005101043

[9] Yokoo T, Fukui A, Matsumoto K, Ohashi T, Sado Y, et al (2008) Generation of transplantable erythropoietin producer derived from human mesenchymal stem cells. Transplantation 85: 1654–1658. 18551074 10.1097/TP.0b013e318173a35d

[10] Aust L, Devlin B, Foster SJ, Halvorsen YD, Hicok K, et al (2004) Yield of human adipose-derived adult stem cells from liposuction aspirates. Cytotherapy 6: 7–14. 14985162 10.1080/14653240310004539

[11] Gonzalez-Cruz RD, Fonseca VC, Darling EM (2012) Cellular mechanical properties reflect the differentiation potential of adipose-derived mesenchymal stem cells. Proc Natl Acad Sci U S A 12: E1523–1529. 10.1073/pnas.1120349109 PMC3386052 22615348

[12] Drewa T, Joachimiak R, Kaznica A, Flisinski M, Brymora A, et al (2008) Bone marrow progenitors from animals with chronic renal failure lack capacity of in vitro proliferation. Transplant Proc 40: 1668–1673. 18589170 10.1016/j.transproceed.2008.03.141

[13] Noh H, Yu MR, Kim HJ, Jeon JS, Kwon SH, et al (2012) Uremia induces functional incompetence of bone marrow-derived stromal cells. Nephrol Dial Transplant 27: 218–225. 21622994 10.1093/ndt/gfr267

[14] Roemeling-van Rhijn M, Reinders ME, de Klein A, Douben H, Korevaar SS, et al (2012) Mesenchymal stem cells derived from adipose tissue are not affected by renal disease. Kidney Int 82: 748–758. 22695328 10.1038/ki.2012.187

[15] Yamada A, Yokoo T, Yokote S, Yamanaka S, Izuhara L, et al (2014) Comparison of multipotency and molecular profile of MSCs between CKD and healthy rats. Hum Cell 27: 59–67. 24496821 10.1007/s13577-013-0082-7

[16] Zuk PA, Zhu M, Mizuno H, Huang J, Futrell JW, et al (2001) Multilineage cells from human adipose tissue: implications for cell-based therapies. Tissue Eng 7: 211–228. 11304456 10.1089/107632701300062859

[17] Banas A, Teratani T, Yamamoto Y, Tokuhara M, Takeshita F, et al (2007) Adipose tissue-derived mesenchymal stem cells as a source of human hepatocytes. Hepatology 46: 219–228. 17596885 10.1002/hep.21704

[18] Dominici M, Le Blanc K, Mueller I, Slaper-Cortenbach I, Marini F, et al (2006) Minimal criteria for defining multipotent mesenchymal stromal cells. The International Society for Cellular Therapy position statement. Cytotherapy 8: 315–317. 16923606 10.1080/14653240600855905

[19] Wagner W, Horn P, Castoldi M, Diehlmann A, Bork S, et al (2008) Replicative senescence of mesenchymal stem cells: a continuous and organized process. PLoS One 3: e2213. 18493317 10.1371/journal.pone.0002213 PMC2374903

[20] Huang J, Gan Q, Han L, Li J, Zhang H, et al (2008) SIRT1 overexpression antagonizes cellular senescence with activated ERK/S6k1 signaling in human diploid fibroblasts. PLoS One 5: e1710. 10.1371/journal.pone.0001710 PMC2249701 18320031

[21] Nekanti U, Dastidar S, Venugopal P, Totey S, Ta M (2010) Increased proliferation and analysis of differential gene expression in human Wharton's jelly-derived mesenchymal stromal cells under hypoxia. Int J Biol Sci 9: 499–512. 10.7150/ijbs.6.499 PMC2945278 20877435

[22] Shimada Y, Nishida H, Nishiyama Y, Kobayashi H, Higuchi T, et al (2011) Proteasome inhibitors improve the function of mutant lysosomal α-glucosidase in fibroblasts from Pompe disease patient carrying c.546G>T mutation. Biochem Biophys Res Commun 415: 274–278. 22027144 10.1016/j.bbrc.2011.10.038

[23] Sahagun G, Moore SA, Fabry Z, Schelper RL, Hart MN (1989) Purification of murine endothelial cell cultures by flow cytometry using fluorescein-labeled griffonia simplicifolia agglutinin. Am J Pathol 134: 1227–32. 2757116 PMC1879935

[24] Basile JR, Holmbeck K, Bugge TH, Gutkind JS (2007) MT1-MMP controls tumor-induced angiogenesis through the release of semaphorin 4D. J Biol Chem 2: 6899–905. 10.1074/jbc.M609570200 17204469

[25] Narita M, Nũnez S, Heard E, Narita M, Lin AW, et al (2003) Rb-mediated heterochromatin formation and silencing of E2F target genes during cellular senescence. Cell 13: 703–16. 10.1016/s0092-8674(03)00401-x 12809602

[26] Okuyama H, Krishnamachary B, Zhou YF, Nagasawa H, Bosch-Marce M, et al (2006) Expression of vascular endothelial growth factor receptor 1 in bone marrow-derived mesenchymal cells is dependent on hypoxia-inducible factor 1. J Biol Chem 281: 15554–15563. 16574650 10.1074/jbc.M602003200

[27] Bastiaansen AJ, Ewing MM, de Boer HC, van der Pouw Kraan TC, de Vries MR, et al (2013) Lysine acetyltransferase PCAF is a key regulator of arteriogenesis. Arterioscler Thromb Vasc Biol 33: 1902–1910. 23788761 10.1161/ATVBAHA.113.301579 PMC4049097

[28] Guedez L, Rivera AM, Salloum R, Miller ML, Diegmueller JJ, et al (2003) Quantitative assessment of angiogenic response by the Directed In Vivo Angiogenesis Assay. American J Pathol 162: 1431–1439. 10.1016/S0002-9440(10)64276-9 PMC1851187 12707026

[29] Xenaki G, Ontikatze T, Rajendran R, Stratford IJ, Dive C, et al (2008) PCAF is an HIF-1alpha cofactor that regulates p53 transcriptional activity in hypoxia. Oncogene 27: 5785–5796. 18574470 10.1038/onc.2008.192 PMC2664615

[30] Ravnskjaer K, Hogan MF, Lackey D, Tora L, Dent SY, et al (2013) Glucagon regulates gluconeogenesis through KAT2B- and WDR5-mediated epigenetic effects. J Clin Invest 123: 4318–4328. 24051374 10.1172/JCI69035 PMC3784539

[31] Ferrari R, Pellegrini M, Horwitz GA, Xie W, Berk AJ, et al (2008) Epigenetic reprogramming by adenovirus e1a. Science 321: 1086–1088. 18719284 10.1126/science.1155546 PMC2693122

[32] Pillai S, Kovacs M, Chellappan S (2010) Regulation of vascular endothelial growth factor receptors by Rb and E2F1: role of acetylation. Cancer Res 70: 4931–4940. 20516113 10.1158/0008-5472.CAN-10-0501 PMC2891185

[33] Lim JH, Lee YM, Chun YS, Chen J, Kim JE, et al (2010) Sirtuin 1 modulates cellular responses to hypoxia by deacetylating hypoxia-inducible factor 1alpha. Mol Cell 38: 864–878. 20620956 10.1016/j.molcel.2010.05.023

[34] Jin Y, Zeng SX, Dai MS, Yang X-J, Lu H (2002) MDM2 inhibits PCAF-mediated p53 acetylation. J Biol Chem 277: 30838–30843. 12068014 10.1074/jbc.M204078200

[35] Linares LK, Kiernan R, Triboulet R, Chable-Bessia C, Latreille D, et al (2007) Intrinsic ubiquitination activity of PCAF controls the stability of the oncoprotein Hdm2. Nat Cell Biol 9: 331–338. 17293853 10.1038/ncb1545

[36] Kelly BD, Hackett SF, Hirota K, Oshima Y, Cai Z, et al (2003) Cell type-specific regulation of angiogenic growth factor gene expression and induction of angiogenesis in nonischemic tissue by a constitutively active form of hypoxia-inducible factor 1. Circ Res 93: 1074–1081. 14576200 10.1161/01.RES.0000102937.50486.1B

[37] Shakib K, Norman JT, Fine LG, Brown LR, Godovac-Zimmermann J (2005) Proteomics profiling of nuclear proteins for kidney fibroblasts suggests hypoxia, meiosis, and cancer may meet in the nucleus. Proteomics 5: 2819–2838. 15942958 10.1002/pmic.200401108

[38] Martínez-Balbás MA, Bauer UM, Nielsen SJ, Brehm A, Kouzarides T (2000) Regulation of E2F1 activity by acetylation. EMBO J 19: 662–671. 10675335 10.1093/emboj/19.4.662 PMC305604

[39] Meyer TW, Hostetter TH (2007) Uremia. N Engl J Med 27: 1316–1325. 10.1056/NEJMra071313 17898101

[40] Bernhardt WM, Schmitt R, Rosenberger C, Munchenhagen PM, Grone HJ, et al (2006) Expression of hypoxia-inducible transcription factors in developing human and rat kidneys. Kidney Int 69: 114–122. 16374431 10.1038/sj.ki.5000062

[41] Bechtel-Walz W, Huber TB (2014) Chromatin dynamics in kidney development and function. Cell Tissue Res 356: 601–608. 24817101 10.1007/s00441-014-1884-y

[42] Chen S, Bellew C, Yao X, Stefkova J, Dipp S, et al (2011) Histone deacetylase (HDAC) activity is critical for embryonic kidney gene expression, growth, and differentiation. J Biol Chem 286: 32775–32789. 21778236 10.1074/jbc.M111.248278 PMC3173185

