# Correction: Adipose Tissue-Derived Mesenchymal Stem Cells in Long-Term Dialysis Patients Display Downregulation of PCAF Expression and Poor Angiogenesis Activation

**DOI:** 10.1371/journal.pone.0157282

**Published:** 2016-06-06

**Authors:** Shuichiro Yamanaka, Shinya Yokote, Akifumi Yamada, Yuichi Katsuoka, Luna Izuhara, Yohta Shimada, Nobuo Omura, Hirotaka James Okano, Takao Ohki, Takashi Yokoo

There is an error in Fig 1. Panel B is a duplicate of Panel A. Please see the corrected Fig 1 below.

**Fig 1:**
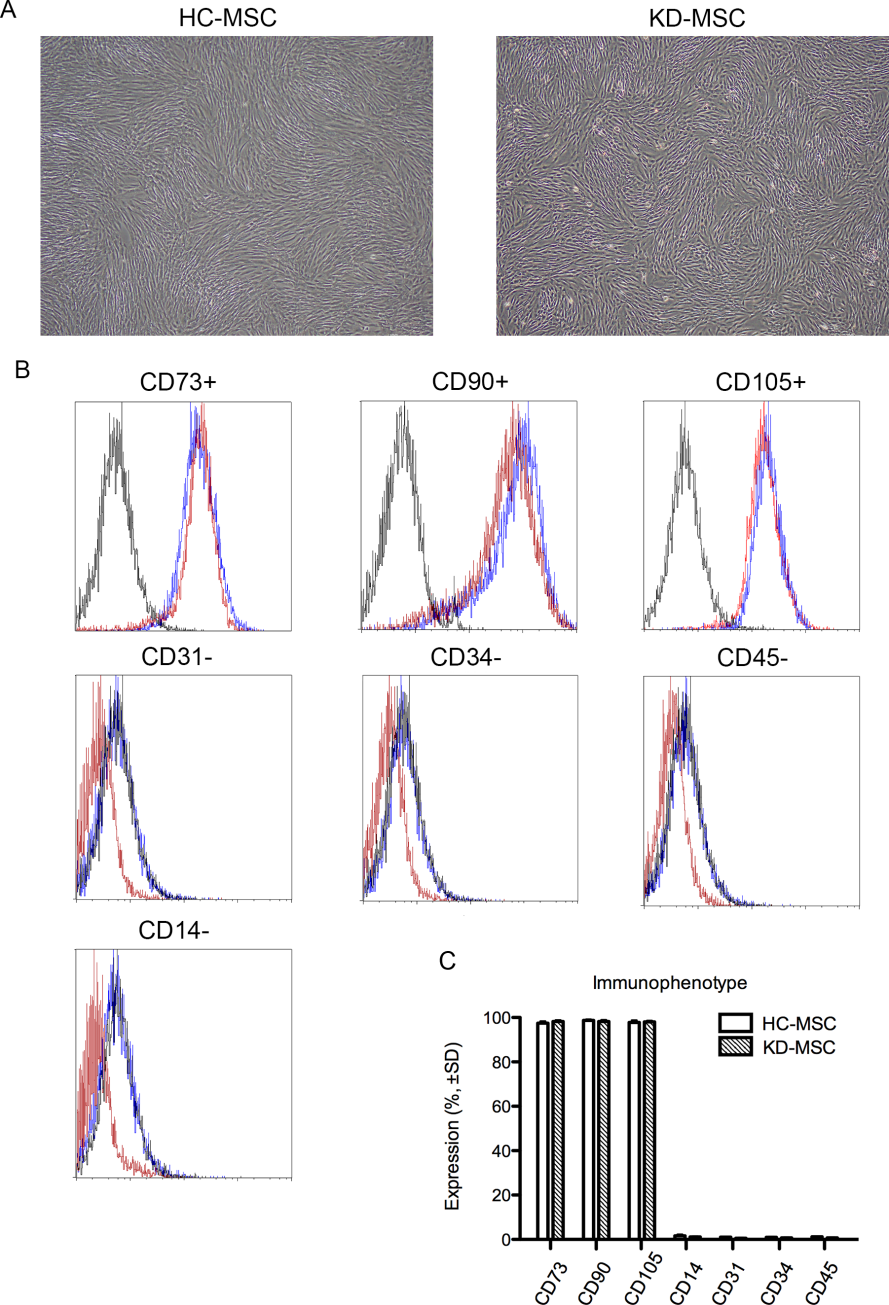
Characteristics of mesenchymal stem cells from healthy controls (HC-MSCs) and patients with ESKD (KD-MSCs). (A) Representative images of HC-MSCs (left) and KD-MSCs (right; original magnification, ×100). (B) Flow cytometric analysis of cell surface marker expression of HC-MSCs (solid lines; *n* = 6) and KD-MSCs (dashed lines; *n* = 9). Isotype-matched IgG controls are represented by solid histograms. (C) Comparison of cell surface marker expression in HC-MSCs (*n* = 6) and KD-MSCs (*n* = 9). The percentages of positive cells are shown. Data are the mean ± SE. There were no significant differences.

There are a number of errors in the caption for Fig 5, “Western blot analysis of PCAF, HIF-1α, and VEGF expression under hypoxia and normoxia.’ Please see the complete, correct Fig 5 caption here.

**Fig 5:**
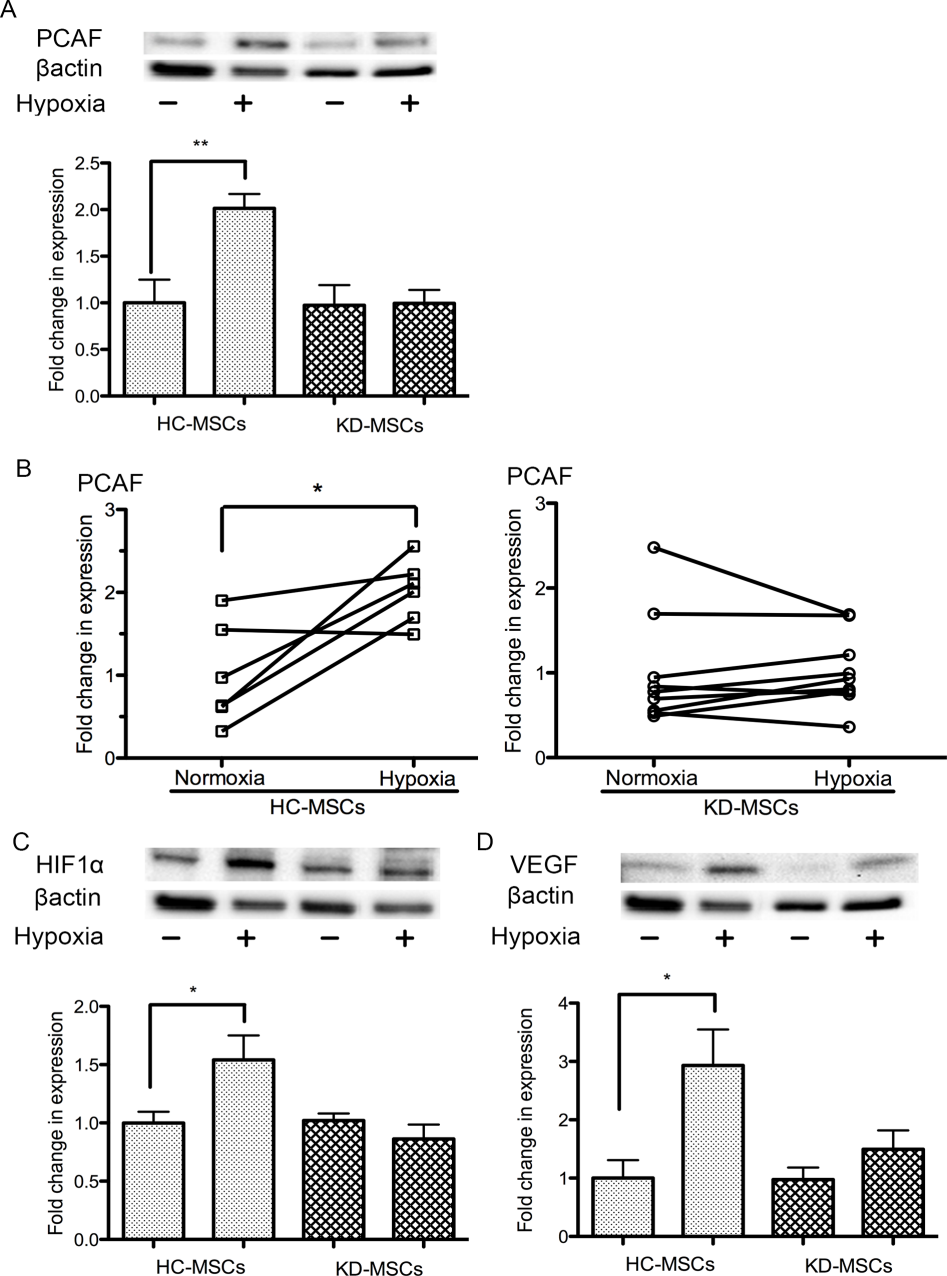
Western blot analysis of PCAF, HIF-1α, and VEGF expression under hypoxia and normoxia. (A) Western blot analysis of PCAF expression in KD-MSCs (*n* = 9) and HC-MSCs (*n* = 6) under normoxia and hypoxia (1% O_2_). Data are the mean ± SE. **P < 0.01 normoxia versus hypoxia in HC-MSCs (two-tailed, unpaired *t*-test). (B) Western blot analysis of PCAF expression at 24 h under hypoxia showed it to be clearly upregulated in HC-MSCs. There was no change in PCAF in KD-MSCs under hypoxia. Data are the mean ± SE (HC-MSCs *n* = 6, KD-MSCs *n* = 9; ^*^P<0.05 versus normoxia, two-tailed, paired *t*-test). (C) Western blot analysis of HIF-1α expression in KD-MSCs (*n* = 9) and HC-MSCs (*n* = 6) under normoxia and hypoxia. Data are the mean ± SE. *P<0.05 versus normoxia (two-tailed, unpaired *t*-test). (D) Western blot analysis of VEGF expression in KD-MSCs (*n* = 9) and HC-MSCs (*n* = 6) under normoxia and hypoxia. Data are the mean ± SE. *P<0.05 versus normoxia (two-tailed, unpaired *t*-test). (A–D) MSC lines were isolated independently. Because of the differing molecular weights of PCAF, HIF1α, VEGF, and β-actin, molecular-weight-bands in the western blot were cut out and stained with their respective specific antibodies. The endogenous β-actin band was used as an internal control for each band derived from other proteins.
